# Synthesis and antifungal activities of novel nicotinamide derivatives containing 1,3,4-oxadiazole

**DOI:** 10.1186/1752-153X-7-64

**Published:** 2013-04-05

**Authors:** Jian Wu, Shenghong Kang, Lijun Luo, Qingcai Shi, Juan Ma, Juan Yin, Baoan Song, Deyu Hu, Song Yang

**Affiliations:** 1 State Key Laboratory Breeding Base of Green Pesticide and Agricultural Bioengineering, Key Laboratory of Green Pesticide and Agricultural Bioengineering, Ministry of Education, Guizhou University, Guiyang, China; 2 Research and Development Center for Fine Chemicals, Guizhou University, Guiyang, 550025, China

## Abstract

**Background:**

Pant diseases in agriculture are extremely difficult to control, which bring about for billions of dollars in economic losses worldwide each year; Nicotinamide derivatives has attracted more and more attention in the field of pesticide due to their broad bioactivities. In an effort to discover new molecules with good antifungal activity, a series of nicotinamide derivatives containing 1,3,4-oxadiazole were synthesized and bio-assayed.

**Results:**

Bioassays demonstrated that some of the title compounds exhibited favorable antifungal activities against *Gibberella zeae*, *Fusarium oxysporum*, and *Cytospora mandshurica*. compounds **7b**, **7c**, **8**, **9a**, **9b**, and **9c** displayed 46.4%, 39.6%, 53.0%, 43.2%, 58.3%, 45.6% activities against *G. Zeae*, respectively; the activities of compounds **7a**, **7b**, **8**, **9a**, **9b**, and **9c** against *F. oxysporum* were 55.2%, 51.1%, 58.9%, 63.2%, 53.3%, and 47.6%, respectively; whereas inhibitory rates of compounds **7a**, **7b**, **7c**, **8**, **9a**, **9b**, and **9c** on *C. mandshurica* were 53.1%, 49.9%, 44.9%, 52.8%, 59.8%, 54.5%, and 49.3%, respectively.

**Conclusion:**

A series of the novel nicotinamide derivatives containing 1,3,4-oxadiazole were synthesized and bio-assayed. The results of antifungal tests revealed that the synthesized nicotinamide derivatives possessed weakly to moderately antifungal activities against *G. zeae*, *F. oxysporum*, and *C. mandshurica*. Most of the synthesized compounds exhibited similar activities as (or higher than) these of hymexozol on their corresponding fungus, and compounds **7a**, **8**, **9a**, and **9b** showed considerable prospects for further optimization. Primary structure-activity relationships revealed that the introduction of the groups of 4-chloro-6-methyl on benzene, the hydrazone group containing *N,N*-dimethylamino, and acetyl at -NH_*2*_ could enhance the antifungal activity.

## Background

In recently years, plant diseases in agriculture are extremely difficult to control due to the continued moist weather during the crop growing season and the resistance of fungicides in the pathogen population, which bring about for billions of dollars in economic losses worldwide each year [1]. Take fusarium head blight (FHB) as example, it was caused by *Gibberella zeae* and was endemic in the wheat producing areas of many provinces of China, this kind of disease have caused an estimated 30 to 50% of reduction and even completely failure of harvests in many wheat-producing areas [2]. Therefore, the controlling of plant pathogens (such as *G. zeae*) has become more and more difficult, and the development of novel fungicidal molecules for these pathogens has been attracted more and more attention.

**Figure 1:**
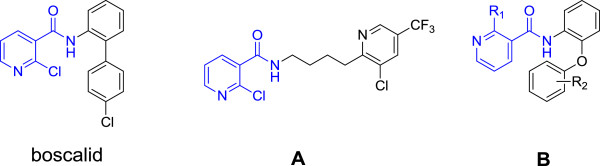
The structures of boscalid and active compounds A and B.

Nicotinamide derivatives, an important class of heterocyclic derivative, has attracted more and more attention in the field of pesticide due to their fungicidal activity [3-5], insecticidal activity [6-9], herbicidal activity [8,10], plant growth regulator activity [11], and bactericidal activity [12-15]. In fungicidal activity regard, some nicotinamide derivatives have been developed and commercialized, an example of such a fungicide is boscalid (Figure 1), which was discovered by BASF and registered in Britain, Germany, and Switzerland in 2004 [16]. In 2008, Coqueron, P Y. *et al.* described a nicotinamide containing 4-(3-chloro-5-(trifluoromethyl)pyridin-2-yl)butyl (**A**, Figure 1 ) possessing good fungicidal activities against *Alternaria alternate*[17]. More recently, Fang W and his co-workers also reported several nicotinamide derivatives (**B**, Figure 1) exhibiting good fungicidal activities after modification of the boscalid structure [18].

1,3,4-oxadiazole derivatives, another important heterocyclic derivative, has attracted more and more attention in development of fungicide, in the past few years, a lots of 1,3,4-oxadiazole derivatives with good antifungal activity have been developed by our group, some of the developed compounds processed good prospects for commercialization [19-24]. New fungicidal molecules are developed in the present work on the basis of the following: incorporation of the sub-structural unit of 1,3,4-oxadiazole into the backbone of boscalid and structural variation by the introduction of different kinds of moiety, resulting in nicotinamides containing a 1,3,4-oxadiazole substructure with broad-spectrum activity. Based on this hypothesis, a series of novel nicotinamide derivatives containing 1,3,4-oxadiazole substructure were designed and synthesized. Biological assays revealed that most of the synthesized compounds were provided with weak to moderate activities against *G. Zeae*, *F. oxysporum*, and *C. mandshurica* at 50 mg/L. Some of the synthesized compounds exhibited similar or higher activities as that of hymexozol on their corresponding fungus, compounds **9a**, **7a**, **8**, and **9b** showed considerable prospects.

## Results and discussion

### Synthesis

**Scheme 1:**
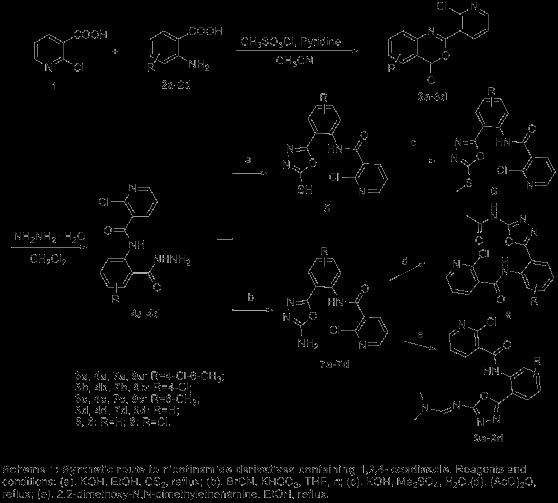
Synthetic route to nicotinamide derivatives containing 1,3,4-oxadiazole. **Synthetic route to nicotinamide derivatives containing 1,3,4-oxadiazole.** Reagents and conditions: (**a**). KOH, EtOH, CS_2_, reflux; (**b**). BrCN, KHCO_3_, THF, rt; (**c**). KOH, Me_2_SO_4_, H_2_O. (**d**). (AcO)_2_O, reflux; (**e**). 2,2-dimethoxy-*N,N*-dimethylethenamine, EtOH, reflux.

**Scheme 2:**
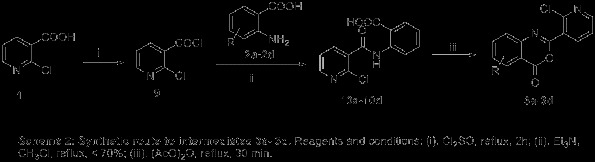
**Synthetic route to intermediates 3a-9c.** Reagents and conditions: (i). Cl_2_SO, reflux, 2 h; (ii). Et_3_N, CH_3_Cl, reflux, <70%; (iii). (AcO)_2_O, reflux, 30 min.

The synthetic protocol of the nicotinamide derivatives containing 1,3,4-oxadiazole are depicted in Scheme 1. Firstly, substituted-2-(2-chloropyridin-3-yl)-4*H*-benzo[*d*] [1,3]oxazin-4-one (**3a**-**3d**) can be synthesized by cyclisation of 2-(2-chloronicotinamido)benzoic acid (**10a**-**10d**) in refluxing acetic anhydride about 30 min (Scheme 2) with excellent yield (>90%) [25-27]. However, the synthetic method showed in Scheme 2 had several shortcomings, which included long steps, poor stability of 2-chloronicotinoyl chloride, low yields and long reaction times for preparation of **10a** to **10d**. A single-step alternative protocol with a short reaction time and high yields (>90%) carried out at room temperature in acetonitrile was thus employed by treatment of 2-chloronicotinic acid (**1**) with 2-amino-sbustitutedbenzoic acid (**2a**-**2d**) in the presence of pyridine and methanesulfonyl chloride [14,25,28,29]. The further reaction of substituted-2-(2-chloropyridin-3-yl)-4*H*-benzo[*d*] [1,3]oxazin-4-one (**3a**-**3d)** with 80% hydrazine hydrate could carry out readily in 1 h at room temperature to give substituted-*N*-(2-(hydrazinecarbonyl) phenyl) nicotinamide (**4a**-**4d)**[14]. Subsequent treatment of intermediates **4d** with CS_2_ in present of KOH in refluxing ethanol afforded 2-chloro-*N*-(2-(5-mercapto-1,3,4-oxadiazol-2-yl)phenyl)nicotinamide (**5**), which further reacted with Me_2_SO_4_ in present of KOH in water to give 2-chloro-*N*-(2-(5-(methylthio)-1,3,4-oxadiazol-2-yl)phenyl)nicotinamide (**6**). Moreover, *N*-(2-(5-amino-1,3,4-oxadiazol-2-yl)-substituted-phenyl)-2-chloronicotinamide **7a-7d** can be prepared by treatment of **4a**-**4d** with BrCN in present of KHCO_3_ in dry tetrahydrofuran (THF), receptivity [30], compound **8** then can be obtained by using compound **7b** in refluxing acetic anhydride for 30 min with 85% yield. Finally, **9a**, **9b**, **9c**, and **9d** were conveniently obtained with >90% yields by treatment of **7a**, **7b**, **7c**, and, **7d** with 2,2-dimethoxy-*N,N*-dimethylethenamine about 15 min in refluxing EtOH, respectively. The procedure for preparation of the intermediates and title compounds can be found in Additional file 1.

The structures of the synthesized compounds (**5**–**9**) were established on the basis of their spectroscopic data. The IR spectra showed absorption bands around near 3230–3400 cm^-1^, 2990–3150 cm^-1^for the N-H, and Ar-H stretching vibrations, respectively. The absorption bands near 1670–1710 cm^-1^ were the presence C = O functional groups, the absorption bands of 1450–1620 cm^-1^ were the skeletal vibration of benzene ring. In the ^1^H-NMR spectra of the title compounds, the -CON*H*Ar proton appeared as a broad singlet at 11.88-10.27 ppm range; the -C*H* = N- proton for compounds **9a**-**9d** mainly appeared as a singlet near the 8.50 ppm; the protons at 6 and 4 positions of pyridine ring were occurred as double-double peaks near 8.57-8.51 and 7.61-8.33, respectively; and the protons at 5 position of pyridine is near the 7.60 ppm. The properties, ^1^H NMR, ^13^C NMR, IR, and the elemental analysis data of the title compounds in more detail are also listed in Additional file 1 [see Additional file 1].

### Antifungal activity and structure activity relationship

The antifungal activities of compounds **5**–**9** against *G. Zeae*, *F. oxysporum*, and *C. mandshurica* were assayed using the poison plate technique [31,32]. Hymexazol and carbendazol were used as positive control. The results listed in Table 1 indicated that most of the synthesized compounds were provided with weak to moderate activities against *G. Zeae*, *F. oxysporum*, and *C. mandshurica* at 50 mg/L. It can be seen that compounds **7b**, **7c**, **8**, **9a**, **9b**, and **9c** displayed 46.4%, 39.6%, 53.0%, 43.2%, 58.3%, 45.6% activities against *G. Zeae*, respectively; the activties of compounds **7a**, **7b**, **8**, **9a**, **9b**, and **9c** against *F. oxysporum* were 55.2%, 51.1%, 58.9%, 63.2%, 53.3%, and 47.6%, respectively; whereas inhibitory rates of compounds **7a**, **7b**, **7c**, **8**, **9a**, **9b**, and **9c** on *C. mandshurica* were 53.1%, 49.9%, 44.9%, 52.8%, 59.8%, 54.5%, and 49.3%, respectively. It can concluted from these active data that these compounds exhibited similar activities as (or higher than) these of hymexozol on their corresponding fungus, and the compounds **9a** (against both *F. oxysporum* and *C. mandshurica*), **7a**, **8**, and **9b** (against *C. mandshurica*) showed considerable prospects.

**Table 1:** Antifungal activities of the title compounds 5–9 at 50 mg/L

**Compound**	**Inhibition rate**^**a**^**(%)**		
	***G. zeae***	***F. oxysporum***	***C.mandshurica***
**5**	17.9 ± 1.6	10.3 ± 0.9	15.5 ± 0.7
**6**	33.2 ± 1.7	21.3 ± 1.1	30.3 ± 1.9
**7a**	19.0 ± 2.9	55.2 ± 0.6	53.1 ± 1.3
**7b**	46.4 ± 1.3	51.1 ± 1.0	49.9 ± 1.1
**7c**	39.6 ± 1.3	31.9 ± 1.4	44.9 ± 0.7
**7d**	27.9 ± 0.2	12.5 ± 1.7	17.3 ± 1.0
**8**	53.0 ± 2.4	58.9 ± 4.3	52.8 ± 1.3
**9a**	43.2 ± 0.6	63.2 ± 0.9	59.8 ± 1.4
**9b**	58.3 ± 2.7	53.3 ± 3.9	54.5 ± 1.2
**9c**	45.6 ± 1.8	47.6 ± 1.0	49.3 ± 1.1
**9d**	35.4 ± 1.9	17.2 ± 0.3	34.3 ± 0.9
Hymexazol^b^	55.54 ± 3.9	56.12 ± 2.1	49.61 ± 3.2
Carbendazol^b^	100 ± 3.74	100 ± 4.9	100 ± 5.23

^a^Average of three replicates, ^b^The commercial agricultural fungicide hymexazol and carbendazol were used for activity comparison.

Primary structure activity relationships (SAR) revealed that antifungal activities on the three fungus can be improved to a certain extent by introduction of *N*,*N*-dimethylamino. For instance, compound **9a** showed much higher activities against both *G. Zeae* and *F. oxysporum* than that of **7a**, and compounds **9b-9d** also showed slightly better activities than these of **7b-7d**. In addition, the introduction of acetyl can enhance the antifungal activity when chlorine was at 4-position of benzene, that why compound **8** showed higher activity than that of compound **7b**. Moreover, the substituent on benzene play an important role when the group on 1,3,4-oxadiazole was “-NH_2_”, as we can see that compound **7a** (R = 4-chloro-6-methyl) showed higher activity than that of **7b** (R = 4-chloro), and compound **7c** (R = 6-methyl) showed slightly lower activity, and the activity of **7d** (R = H) was the lowest one. So the active order was **7a > 7b > 7c > 7d**; a similar case can be found when -NH_2_ group was changed to –N = CHN (CH_3_)_2_ (**9a > 9b > 9c > 9d**). Finally, the compound with thiol (**5**) displayed much lower activity than that of the compound with amino group, but the introduction of -CH_3_ group (compound **6**) could increase the activity against all the test fungus. Further structural modification is currently underway basing on the primary SAR to discover the potential nicotinamides containing 1,3,4-oxadiazole.

## Experimental

### Chemistry

Unless otherwise stated, all the reagents and reactants were purchased from commercial suppliers; melting points were uncorrected and determined on a XT-4 binocular microscope (Beijing Tech Instrument Co., China). The ^1^H-NMR and ^13^C-NMR spectra were recorded on a JEOL ECX 500 NMR spectrometer (JEOL Ltd., Japan) at room temperature operating at 500 MHz for ^1^H-NMR and 125 MHz for ^13^C-NMR by using CDCl_3_ or DMSO as solvents and TMS as an internal standard; infrared spectra were recorded in KBr on a IR Pristige-21 spectrometer (Shimadzu corporation, Japan); elemental analysis was performed on an Elemental Vario-III CHN analyzer (Elementar, German). The course of the reactions was monitored by TLC; analytical TLC was performed on silica gel GF 254. Intermediates **3** and **4** were prepared according to the reported methods [14] and used without further purifications, the process for preparing of them can be found in Additional file 1.

### Antifungal biological assay

All the compounds (**5**, **6**, **7a-7d**, **8**, **9a-9d)** were evaluated for their antifungal activities against *G. zeae*, *F. oxysporium*, and *C. mandshurica in vitro* as described in literature [31,32]. The results of preliminary bioassays were compared with the experimental data of the commercial agricultural fungicide, hymexazol and carbendazim. All the compounds were dissolved in dimethyl sulfoxide (DMSO, 10 mL) before mixing with potato dextrose agar (PDA, 90 mL). The compounds were tested at a concentration of 50 mg/L. All fungal species were incubated in PDA at 27 ±1°C for 5 days to obtain new mycelium for antifungal assay. Mycelia dishes approximately 4 mm in diameter were cut from the culture medium. One of the specimens was picked up with a sterilized inoculation needle and then inoculated in the center of the PDA plate aseptically. The inoculated plates were incubated at 27 ±1°C for 5 days. DMSO in sterile distilled water served as the control, whereas hymexazol and carbendazim acted as the positive control. Three replicates were conducted for each treatment. The radial growth of the fungal colonies was measured on the sixth day and the data were statistically analyzed. The *in vitro* inhibiting effects of the test compounds on the fungi were calculated by the formula:

I(%) = [(C-T)/(C-0.4)] × 100, where C represents the diameter of fungal growth on untreated PDA, T represents the diameter of fungi on treated PDA, and I is the inhibitory rate.

## Conclusion

In conclusion, a series of the novel nicotinamide derivatives containing 1,3,4-oxadiazole were designed and synthesized. The synthesized compounds were characterized by spectral data (^1^H NMR, ^13^C NMR, IR) and elemental analysis. All of the compounds were subjected to fungicidal activities *in vitro* against *G. zeae*, *F. oxysporum*, and *C. mandshurica*. The results showed that the synthesized nicotinamide derivatives possessed weakly to moderately antifungal activities against the tested fungus. Most of the synthesized compounds exhibited similar activities as (or higher activities than) that of hymexozol on their corresponding fungus, and the compounds **9a**, **7a**, **8**, and **9b** showed considerable prospects for further optimization. Primary structure activity relationships revealed that the introduction of the groups of 4-chloro-6-methyl on benzene, the hydrazone group containing *N,N*-dimethylamino, and acetyl at -NH_2_ could enhance the antifungal activities against *G. zeae*, *F. oxysporum*, and *C. mandshurica*. However, the structures of the synthesized compounds need to be optimized. Further structural modification and biological evaluation are currently underway to explore the full potential of this kind of nicotinamides containing 1,3,4-oxadiazole.

## Competing interests

The authors declare that they have no competing interests.

## Authors’ contributions

The current study is an outcome of constructive discussion with BAS, SY and DYH; JW carry out their synthesis and characterization experiments was also involved in the drafting of the manuscript. JY performed the antifungal activities; JM and QC Shi carried out the ^1^H NMR, ^13^C NMR spectral analyses; SHK and LJL carried out the elemental analysis and infrared spectroscopy. JW and SY were involved in revising the manuscript. All authors read and approved the final manuscript.

## Supplementary Material

Additional file 1 **Experimental details and spectroscopic data of the intermediates 3–4 and the title compounds 5**–**9. **Which includes the experimental procedure and the ^1^H NMR of intermediates (**3a-3d** and **4a-4d)**, the experimental protocols and the spectroscopic data of title compounds **5, 6, 7a-7a, 8**, and **9a-9d**. All the copies of ^1^H NMR and ^13^C NMR for the title compounds were also presented in this additional file. Click here for file
