# Supplementary material for: Synthesis and antifungal activities of novel nicotinamide derivatives containing 1,3,4-oxadiazole
Source: Chem Cent J. 2013 Apr 5;7:64. doi: 10.1186/1752-153X-7-64 (PMC3621414; doi:10.1186/1752-153X-7-64)

Additional file 1

**Synthesis and Antifungal Activities of novel Nicotinamide Derivatives containing 1,3,4-oxadiazole**

Jian Wu*, Shenghong Kang, Lijun Luo, Qingcai Shi, Juan Ma, Juan Yin, Baoan Song, Deyu Hu, Song Yang

Address: State Key Laboratory Breeding Base of Green Pesticide and Agricultural Bioengineering, Key Laboratory of Green Pesticide and Agricultural Bioengineering, Ministry of Education, Research and Development Center for Fine Chemicals, Guizhou University, Guiyang 550025, China.

Author to whom correspondence should be addressed;

Tel.: +86 851 829 2090; Fax: +86 829 2090.

E-Mail: JW: [wujian2691@126.com](mailto:wujian2691@126.com)

KSH: [concenhom0627@126.com](mailto:concenhom0627@126.com)

LJL:luolijun01013610@163.com

QCS: [shiqingcai1988@yahoo.com.cn](mailto:shiqingcai1988@yahoo.com.cn)

JM: [majuanmalong@163.com](mailto:majuanmalong@163.com)

JY: [yinjuan2009292018@126.com](mailto:yinjuan2009292018@126.com)

BAS: [basong@gzu.edu.cn](mailto:basong@gzu.edu.cn)

DYH: [fcc.dyhu@gzu.edu.cn](mailto:fcc.dyhu@gzu.edu.cn)

SY: [yangsdqj@126.com](mailto:yangsdqj@126.com)

**Supporting Information**

**Experimental Procedure**

***General Synthetic Procedure for intermediates 3a-3d***

To a mixture of 2-chloronicotinic acid (21.56 mmoL) and 2-amino-substitutedbenzoic acid (21.56 mmoL) in acetonitrile (30 mL) was slowly added a solution of pyridine (10 mL) and methyl sulfonyl chloride (10 mL) at less than 5 ^o^C. The resulting mixture was warmed to room temperature and stirred for 5 h. When the reaction was completed, the mixture was transferred to a 500 mL beaker, with solution of saturated Na_2_CO_3_ adjusted to pH=7, then filtered to obtain a light yellow solid. The ^1^H NMR for **3a**, **3b**, **3c** and **3d** were listed as follows, respectively.

**3a**, Light yellow solid; yield, 93.1%; m.p: 185-187 ^o^C. ^1^H-NMR (500 MHz, CDCl_3_): 8.57 (dd, 1H, ^4^*J*_HH_=2.25 Hz, ^3^*J*_HH_=4.55 Hz, 4-pyridine-H), 8.35 (dd, 1H, ^4^*J*_HH_=1.70 Hz, ^3^*J*_HH_=7.45 Hz, 6-pyridine-H), 8.08 (d, 1H, *J*_HH_=2.30 Hz, 3-pyridine-H), 7.68 (d, 1H, *J*=2.30 Hz, 6-Ph-H), 7.36 (dd, 1H, ^4^*J*_HH_=4.60 Hz, ^3^*J*_HH_=8.05 Hz, 4-Ph-H), 2.63 (s, 3H, Ph-CH_3_).

**3b**, yield 92.15%; m.p: 167-168 ^o^C. ^1^H-NMR (500 MHz, CDCl_3_): 8.63 (dd, 1H, ^4^*J*_HH_=1.70 Hz, ^3^*J*_HH_=4.55 Hz, 6-pyridine-H), 8.55 (d, 1H, ^3^*J*_HH_=8.6 Hz, Ph-H ), 8.28 (dd, 1H, ^4^*J*_HH_=1.15 Hz, ^3^*J*_HH_=7.45 Hz, 4-pyridine-H), 7.70 (d, 1H, ^4^*J*_HH_=2.3 Hz, Ph-H ), 7.62~7.64 (m, 2H, 5-pyridine-H, Ph-H).

**3c,** Light yellow solid; yield, 77.2%; m.p.: 178-179 ^o^C; ^1^H-NMR (CDCl_3_, 500 MHz): *δ* 8.57 (dd, ^3^*J* = 2.30 Hz, ^4^*J* = 5.15 Hz, 1H, 6-Pyridine-H), 8.36 (dd, ^3^*J* = 1.70 Hz, ^4^*J* = 7.40 Hz, 1H, 4-Pyridine-H), 8.12 (d, *J* = 6.85 Hz, 1H, Ph-H), 7.73 (d, *J* = 6.90 Hz, 1H, Ph-H), 7.50 (t, *J* = 8.05 Hz, 1H, Ph-H), 7.44 (dd, ^3^*J* = 4.55 Hz, ^4^*J* = 7.45 Hz, 1H, 5-Pyridine-H), 2.65 (s, 3H, Ar-CH_3_).

**3d**, yellow solid; yield, 77.2%; m.p.: 168-170 ^o^C; ^1^H-NMR (CDCl_3_, 500 MHz): *δ* 8.49 (dd, 1H, ^4^*J*_HH_=2.30 Hz, ^3^*J*_HH_=5.15 Hz, 6-pyridine-H), 8.10-8.13 (m, 2H, 4-pyridine-H + Ph-H), 7.89 (d, 1H, ^3^*J*_HH_=8.0 Hz, Ph-H), 7.46 (t, 1H, ^3^*J*_HH_=8.05 Hz, Ph-H), 7.34 (dd, 1H, ^4^*J*_HH_=4.60 Hz, ^3^*J*_HH_=7.45 Hz, 5-pyridine-H), 7.25 (t, 1H, ^3^*J*_HH_=7.45 Hz, Ph-H).

**General Synthetic Procedure for intermediates 4a-4d**

A mixture of intermediates **3a, 3b, 3c**, and **3d** (10 mmol) in THF (20 mL) was slowly added to 80% hydrazine hydrate (12 mmol) at room temperature. After stirring for 5-10 min, the reaction mixture quickly precipitated white solid. Then resulting solution was kept stirring for another 5 h at room temperature, and filtered to obtain intermediates **4a**, **4b**, **4c** and **4d**, respectively. The ^1^H NMR for **4a**, **4b**, **4c** and **4d** were listed as follows, respectively.

**4a**, white solid; yield, 84.5%; m.p. 231-233 ^o^C; ^1^H-NMR (500 MHz, DMSO-*d*_6_): *δ* 10.12 (br s, 1H, -CON*H*Ar), 9.55 (s, 1H, -CON*H*N), 8.52 (dd, 1H, ^4^*J*_HH_=1.75 Hz, ^3^*J*_HH_=4.15 Hz, 4-pyridine-H), 8.05 (dd, 1H, ^4^*J*_HH_=1.70 Hz, ^3^*J*_HH_=7.40 Hz, 6-pyridine-H), 7.62 (dd, 1H, ^4^*J*_HH_=4.60 Hz, ^3^*J*_HH_=8.55 Hz, 3-pyridine-H), 7.46 (d, 1H, *J*_HH_=2.30 Hz, 6-Ph-H), 7.23 (d, 1H, *J*=2.90 Hz, 4-Ph-H), 4.42(br s, 2H, -NH_2_), 2.31 (s, 3H, Ph-CH_3_).

**4b**, white solid; yield, 91%; m.p. 225-227 ^o^C; ^1^H-NMR (500 MHz, DMSO-*d*_6_): *δ* 10.54 (br s, 1H, -CON*H*Ar), 9.65 (br s, 1H, -CON*H*N), 8.45 (dd, 1H, ^4^*J*_HH_=1.75 Hz, ^3^*J*_HH_=4.15 Hz, 4-pyridine-H), 8.12 (dd, 1H, ^4^*J*_HH_=1.70 Hz, ^3^*J*_HH_=7.40 Hz, 6-pyridine-H), 7.89 (d, 1H, ^3^*J*_HH_=8.6 Hz, Ph-H ), 7.55 (dd, 1H, ^4^*J*_HH_=1.15 Hz, ^3^*J*_HH_=7.45 Hz, 4-pyridine-H), 7.60 (d, 1H, ^4^*J*_HH_=2.3 Hz, Ph-H ), 7.45~7.48 (m, 2H, 5-pyridine-H, Ph-H), 4.45(br s, 2H, -NH_2_).

**4c**,White solid; yield, 80.4%; m.p.: 203-204 ^o^C; ^1^H-NMR (DMSO-*d_6_*, 500 MHz): *δ* 10.11 (s, 1H, -CONH-), 9.46(s, 1H, -CON*H*NH_2_-), 8.52 (dd, ^3^*J* = 2.30 Hz, ^4^*J* = 5.15 Hz, 1H, 6-Pyridine-H), 8.07 (dd, ^3^*J* = 1.75 Hz, ^4^*J* = 7.45 Hz, 1H, 4-Pyridine-H), 7.59 (dd, ^3^*J* = 4.55 Hz, ^4^*J* = 7.45 Hz, 1H, 5-Pyridine-H), 7.41 (dd, ^3^*J* = 1.70 Hz, ^4^*J* = 9.15 Hz, 1H, Ph-H), 7.31 ~ 7.26 (m, 2H, Ph-H), 4.41 (s, 2H, -NH_2_), 2.31 (s, 3H, Ar-CH_3_).

**4d**,White solid; yield, 89.4 %; m.p.: 189 ~ 191 ^o^C; ^1^H-NMR (DMSO-*d_6_*, 500 MHz): *δ* 10.54 (s, 1H, -CONH-), 9.65 (s, 1H, -CON*H*NH_2_-), 8.52 (dd, ^3^*J* = 2.30 Hz, ^4^*J* = 5.15 Hz, 1H, 6-Pyridine-H), 8.07 (dd, ^3^*J* = 1.75 Hz, ^4^*J* = 7.45 Hz, 1H, 4-Pyridine-H), 7.67-7.69 (m, 2H, 4-pyridine-H + Ph-H), 7.55 (d, 1H, ^3^*J*_HH_=8.0 Hz, Ph-H), 7.46 (t, 1H, ^3^*J*_HH_=8.05 Hz, Ph-H), 7.47 (dd, 1H, ^4^*J*_HH_=4.60 Hz, ^3^*J*_HH_=7.45 Hz, 5-pyridine-H), 7.34 (t, 1H, ^3^*J*_HH_=7.45 Hz, Ph-H), 4.47 (s, 2H, -NH_2_).

**The synthesis, physical and spectral data for the title compounds 5, 6, 7a-7d, 8 and 9a-9d are listed below.**

*Synthesis of 2-chloro-N-(2-(5-mercapto-1,3,4-oxadiazol-2-yl)phenyl)nicotinamide* **(5)**. To a well stirred solution of **4d** (1.53 g) and CS_2_ (1.0 g) in EtOH (5 mL), KOH (1 g) in EtOH (8 mL) was slowly added at room temperature. After completion of the addition, the result mixture was refluxed for 3 h and concentrated under vacuum to afford a yellow solid. 10 mL of H_2_O was then added and adjusted to pH=7 using 5% HCl to afford yellow solid, which was ﬁltered off and recrystallized from ethanol to give compound **5**. White solid, yield, 85%; m.p. 254-255 ^o^C; ^1^H-NMR (500 MHz, DMSO-*d*_6_): *δ* 10.62 (br s, 1H, -CH_3_CON*H*- ), 8.52 (dd, 1H, ^4^*J*_HH_=1.75 Hz, ^3^*J*_HH_=7.45 Hz, 6-pyridine-H), 8.11 (dd, 1H, ^4^*J*_HH_=1.75 Hz, ^3^*J*_HH_=7.45 Hz, 4-pyridine-H), 7.80 (d, 1H, ^3^*J*_HH_=6.90 Hz, Ph-H), 7.74 (d, 1H, ^3^*J*_HH_=8.00 Hz, Ph-H), 7.66 (t, 1H, ^3^*J*_HH_=7.45 Hz, Ph-H), 7.56 (dd, 1H, ^4^*J*_HH_=5.15 Hz, ^3^*J*_HH_=8.6 Hz, 5-pyridine-H), 7.40 (t, 1H, ^3^*J*_HH_=7.45 Hz, Ph-H). ^13^C-NMR (125 MHz, DMSO) *δ*: 177.65, 164.22, 160.18, 151.48, 147.14, 138.57, 135.86, 133.24, 132.85, 129.72, 126.82, 125.95, 123.71, 117.30. IR (KBr, cm^-1^): *ν* 3357, 3072, 2914, 1689, 1612, 1600, 1581, 1548, 1517, 1348, 1317, 1348, 1303, 1116; *anal. calc.* for C_14_H_9_ClN_4_O_2_S: C 50.53%, H 2.73%, N 16.84%; found: C 50.66%, H 2.85%, N 17.19%.

*Synthesis of* *2-chloro-N-(2-(5-(methylthio)-1,3,4-oxadiazol-2-yl)phenyl)nicotinamide* **(6).** To a mixture of compound **5** (0.3 g) in solution of 5% NaOH, dimethyl sulfate (2 mL) was slowly added at room temperature, the results mixture was then stirred for another 2 h to give white solid, which was then ﬁltered off and recrystallized from ethanol to give compound **6**; white solid, yield, 87.5%; m.p. 164-165 ^o^C; ^1^H-NMR (500 MHz, DMSO-*d*_6_): *δ* 10.88 (br s, 1H, -CH_3_CON*H*-), 8.54 (dd, 1H, ^4^*J*_HH_=1.7Hz, ^3^*J*_HH_=5.15 Hz, 6-pyridine -H), 8.13-8.17 (m, 2H, 4-pyridine-H + Ph-H), 7.92 (d, 1H, ^3^*J*_HH_=8.0 Hz, Ph-H), 7.65 (t, 1H, ^3^*J*_HH_=8.05 Hz, Ph-H), 7.59 (dd, 1H, ^4^*J*_HH_=4.60 Hz, ^3^*J*_HH_=7.45 Hz, 5-pyridine-H), 7.39 (t, 1H, ^3^*J*_HH_=7.45 Hz, Ph-H), 2.70 (s, 3H, SCH_3_). ^13^C-NMR (125 MHz, DMSO) *δ*: 171.63, 165.18, 164.61, 164.20, 164.03, 151.69, 147.08, 138.80, 136.37, 133.11, 132.82, 129.40, 129.29, 126.08, 123.89, 115.29, 14.79. IR (KBr, cm^-1^): *ν* 3250, 3190, 3025, 1678, 1620, 1598, 1581, 1543, 1487, 1471, 1438, 1319, 1192; *anal. calc.* for C_15_H_11_ClN_4_O_2_S: C 51.95%, H 3.20%, N 16.16%; found: C 51.98%, H 3.14%, N 15.83%.

***General Synthetic Procedure for title compounds 7a-7d***

To a well stirred solution of **4** (**4a**-**4d**) (2.00 mmol) in THF (8 mL) anhydrous sodium bicarbonate (0.5 g) and cyanogen bromide (2.00 mmol) were added. The mixture was stirred at room temperature and monitored by TLC. After completion of the reaction, the solvent was evaporated, results mixture was then added in 10 mL of water to give a white solid, which was then ﬁltered off and purified by recrystallizing from ethanol to give compound title compounds **7a-7d**. The data for **7a**-**7d** are listed as follows.

*Data for N-(2-(5-amino-1,3,4-oxadiazol-2-yl)-4-chloro-6-methylphenyl)-2-chloro- nicotinamide* **(7a).** White solid; yield, 89%; m.p. 270-271 ^o^C; ^1^H-NMR (500 MHz, DMSO-*d*_6_): *δ* 10.38 (br s , 1H, -CON*H*Ar), 8.53 (dd, 1H, ^4^*J*_HH_=2.30 Hz, ^3^*J*_HH_=4.60 Hz, 6-pyridine-H), 8.19 (dd, 1H, ^4^*J*_HH_=2.30 Hz, ^3^*J*_HH_=7.45 Hz, 4-pyridine-H), 7.60~7.62 (m, 3H, 5-pyridine-H, 2Ph-H, -N=C*H*), 7.33 (br s, 1H, -NH_2_), 2.36 (s, 3H, Ph-CH_3_). ^13^C-NMR (125 MHz, DMSO) *δ*: 163.49, 154.71, 150.22, 146.26, 139.62, 137.86, 132.53, 131.53, 131.25, 124.82, 123.80, 122.78, 17.68. IR (KBr, cm-1): ν 3321, 3132, 3061, 1691, 1664, 1677, 1494, 1402, 1294, 1139; *anal. calc.* for C_15_H_11_Cl_2_N_5_O_2_: C 49.47%, H 3.04%, N 19.23%; found: C 49.48%, H 3.02%, N 19.33%.

*Data for N-(2-(5-amino-1,3,4-oxadiazol-2-yl)-4-chlorophenyl)-2-chloronicotinamide* **(7b).** White solid; yield, 92%; m.p. 274-276 ^o^C; ^1^H-NMR (500 MHz, DMSO-*d*_6_): *δ* 11.22 (br s , 1H, -CON*H*Ar), 8.61 (dd, 1H, ^4^*J*_HH_=1.70 Hz, ^3^*J*_HH_=4.55 Hz, 6-pyridine-H), 8.52 (d, 1H, ^3^*J*_HH_=8.6 Hz, Ph-H ), 8.22 (dd, 1H, ^4^*J*_HH_=1.15 Hz, ^3^*J*_HH_=7.45 Hz, 4-pyridine-H), 7.70 (d, 1H, ^4^*J*_HH_=2.3 Hz, Ph-H ), 7.63~7.67 (m, 2H, 5-pyridine-H, Ph-H), 7.54 (br s, 2H, -NH). ^13^C-NMR (125 MHz, DMSO) *δ*: 164.16, 163.89, 156.05, 151.93, 147.04, 138.99, 135.04, 132.62, 131.24, 128.90, 126.58, 124.01, 123.60, 115.61. IR (KBr, cm^-1^): *ν* 3321, 3242, 3140, 3072, 1675, 1614, 1597, 1537, 1402, 1317, 1259, 1072, 1043; *anal. calc.* for C_14_H_9_Cl_2_N_5_O_2_: C 48.02%, H 2.59%, N 20.00%; found: C 47.98%, H 2.67%, N 19.78%.

*Data for N-(2-(5-amino-1,3,4-oxadiazol-2-yl)-6-methylphenyl)-2-chloronicotinamide* **(7c).** White solid; yield, 94%; m.p. 255-257 ^o^C; ^1^H-NMR (500 MHz, DMSO-*d*_6_): *δ* 10.27 (br s , 1H, -CON*H*Ar), 8.50 (dd, 1H, ^4^*J*_HH_=1.75 Hz, ^3^*J*_HH_=2.85 Hz, 6-pyridine-H), 8.18 (dd, 1H, ^4^*J*_HH_=1.70 Hz, ^3^*J*_HH_=7.45 Hz, 4-pyridine-H), 7.56-7.58 (m, 2H, 5-pyridine-H + Ph-H), 7.45 (d, 1H, *J*_HH_=7.40 Hz, Ph-H), 7.36 (t, 1H, *J*_HH_=7.40 Hz, Ph-H), 7.20 (br s, 2H, -NH_2_), 2.33 (s, 3H, -CH_3_). ^13^C-NMR (125 MHz, DMSO) *δ*: 164.13, 156.68, 150.93, 147.12, 138.70, 137.87, 133.63, 133.38, 132.89, 128.06, 126.38, 123.59, 123.08, 18.69. IR (KBr, cm^-1^): *ν* 3267, 3132, 3084, 1666, 1622, 1583, 1514, 1408, 1361, 1298, 1049, 997, 833; *anal. calc.* for C_15_H_11_ClN_5_O_2_: C 54.64%, H 3.67%, N 21.24%; found: C 54.48%, H 3.72%, N 20.99%.

*Data for N-(2-(5-amino-1,3,4-oxadiazol-2-yl)phenyl)-2-chloronicotinamide* **(7d)** White solid; yield, 88%; m.p. 245-246 ^o^C; ^1^H-NMR (500 MHz, DMSO-*d*_6_): *δ* 11.25 (br s , 1H, -CON*H*Ar), 8.58 (dd, 1H, ^4^*J*_HH_=1.75 Hz, ^3^*J*_HH_=5.15 Hz, 6-pyridine-H), 8.49 (d, 1H, ^3^*J*_HH_=8.6 Hz, Ph-H), 8.22 (dd, 1H, ^4^*J*_HH_=1.70 Hz, ^3^*J*_HH_ = 8.00 Hz, Ph-H), 7.74 (dd, 1H, ^4^*J*_HH_=1.70 Hz, ^3^*J*_HH_=7.45 Hz, 4-pyridine-H), 7.62 (dd, 1H, ^4^*J*_HH_=5.15 Hz, ^3^*J*_HH_=8.00 Hz, 5-pyridine-H), 7.57 (t, 1H, *J*_HH_=7.45 Hz, Ph-H), 7.45 (br s, 2H, -NH_2_), 7.34 (t, 1H, *J*_HH_=7.45 Hz, Ph-H). ^13^C-NMR (125 MHz, DMSO) *δ*: 157.18, 151.81, 147.02, 138.95, 136.29, 132.91, 131.70, 127.30, 125.33, 124.03, 121.72, 113.75. IR (KBr, cm^-1^): *ν* 3334, 3261, 3140, 2920, 1672, 1620, 1598, 1577, 1548, 1440, 1398, 1325, 1118, 1070, 1039, 894; *anal. calc.* for C_14_H_10_ClN_5_O_2_: C 53.26%, H 3.19%, N 22.18%; found: C 53.76%, H 3.68%, N 21.89%.

*Synthesis of* *N-(2-(5-acetamido-1,3,4-oxadiazol-2-yl)-4-chlorophenyl)-2-chloronic otinamide*(**8**). To 4 mL of acetic anhydride, 1 mmol of *N*-(2-(5-amino-1,3,4-oxadiazol-2-yl)-4-chlorophenyl)-2-chloronicotinamide (**7b**) was added, the result mixture was then stirred at refluxing and monitored by TLC, after reacting for 30 min, the result mixture was added to water (10 mL) to afford white solid, which was then ﬁltered off and recrystallized from ethanol to give compound title compound **8**. white solid; yield, 85%; m.p. 218-220 ^o^C; ^1^H-NMR (500 MHz, DMSO-*d*_6_): *δ* 11.88 (br s, 1H, -CH_3_CON*H*- ), 10.95 (br s , 1H, -CON*H*Ar), 8.55 (dd, 1H, ^4^*J*_HH_=2.30 Hz, ^3^*J*_HH_=4.60 Hz, 6-pyridine-H), 8.18 (d, 1H, *J*_HH_=8.6 Hz, Ph-H), 8.21 (d, 1H, ^4^*J*_HH_=2.30 Hz, ^3^*J*_HH_=4.60, 4-pyridine-H), 7.80 (s, 1H, Ph-H), 7.67 (d, 1H,*J*_HH_=9.15 Hz, Ph-H), 7.62-7.63 (m, 1H, 5-pyridine-H), 2.17 (s, 3H, -CH_3_). ^13^C-NMR (125 MHz, DMSO) *δ*: 164.13, 156.68, 150.93, 147.12, 138.70, 137.87, 133.63, 133.38, 132.89, 128.06, 126.38, 123.59, 123.08, 18.69. IR (KBr, cm^-1^): *ν* 3242.3, 3174, 3107, 3064, 2920, 1708, 1685, 1614, 1575, 1541, 1487, 1398, 1323, 1265; *anal. calc.* for C_16_H_11_Cl_2_N_5_O_3_: C 49.00%, H 2.83%, N 17.86%; found: C 48.76%, H 3.03%, N 17.48%.

***General Synthetic Procedure for title compounds 9a-9d.***

To a well stirred solution of **7** (**7a**-**7d**) (1.00 mmol) in ethanol (5 mL) 1 mL of 2,2-dimethoxy-*N,N*-dimethylethenamine (1 mL) were added. The mixture was stirred at refluxing temperature and monitored by TLC. After completion of the reaction, the solvent was evaporated, results mixture was then added in 10 mL of water to give a white solid, which was then ﬁltered off and purified by recrystallizing from ethanol to give compound title compounds **9a-9d**. The data for **9a**-**9d** are listed as follows.

*Data for 2-chloro-N-(4-chloro-2-(5-(((dimethylamino)methylene)amino)-1,3,4-oxad iazol-2-yl)-6-methylphenyl)nicotinamide* (**9a**). White solid; yield, 92%; m.p. 244-246 ^o^C.^1^H-NMR (500 MHz, DMSO-*d*_6_): *δ* 10.45 (br s , 1H, -CON*H*Ar), 8.53-8.57 (m, 2H, 6-pyridine-H + Ph-H), 8.30 (dd, 1H, ^4^*J*_HH_=1.70 Hz, ^3^*J*_HH_=7.45 Hz, 4-pyridine-H), 7.83 (s, 1H, -N=CH), 7.59~7.61 (m, 2H, 5-pyridine-H, Ph-H), 3.18 (s, 3H, -NCH_3_), 3.07 (s, 3H, -NCH_3_), 2.38 (s, 3H, -NCH_3_). ^13^C-NMR (125 MHz, DMSO) *δ*: 168.13, 164.31, 159.12, 158.40, 150.85, 147.09, 140.41, 138.53, 132.44, 125.74, 124.36, 123.47, 41.03, 34.94, 18.66. IR (KBr, cm^-1^): ν 3458, 3275, 2990, 1680, 1631, 1566, 1506, 1473, 1402, 1365, 1300; *anal. calc.* for C_18_H_16_Cl_2_N_6_O_2_: C 51.56%, H 3.85%, N 20.04%; found: C 41.32%, H 3.47%, N 20.23%.

*Data for 2-chloro-N-(4-chloro-2-(5-(((dimethylamino)methylene)amino)-1,3,4-oxad iazol-2-yl)phenyl)nicotinamide***(9b).** White solid; yield, 93%; m.p. 237-238 ^o^C.^1^H-NMR (500 MHz, DMSO-*d*_6_): *δ* 11.30 (br s, 1H, -CH_3_CON*H*- ), 8.57 (dd, 1H, ^4^*J*_HH_=2.30 Hz, ^3^*J*_HH_=4.55 Hz, 6-pyridine-H), 8.54 (s, 1H, =CH), 8.47 (d, 1H, *J*_HH_=8.6 Hz, Ph-H), 8.22 (dd, 1H, ^4^*J*_HH_=2.30 Hz, ^3^*J*_HH_=7.45 Hz, 4-pyridine-H), 7.94 (d, 1H, ^4^*J*_HH_=2.85 Hz, Ph-H), 7.66 (dd, 1H, ^4^*J*_HH_=2.30 Hz, ^3^*J*_HH_=8.6 Hz, Ph-H), 7.61 (dd, 1H, ^4^*J*_HH_=5.15 Hz, ^3^*J*_HH_=7.45 Hz, 5-pyridine-H), 3.15 (s, 3H, -CH_3_), 3.00 (s, 3H, -CH_3_). ^13^C-NMR (125 MHz, DMSO) *δ*: 170.62, 168.68, 167.27, 164.18, 163.31, 159.55, 158.54, 151.94, 147.06, 138.97, 135.32, 131.69, 129.15, 127.22, 123.96, 41.13, 35.06. IR (KBr, cm^-1^): *ν* 3300, 3066, 2919, 1687, 1639, 1595, 1560, 1533, 1487, 1396, 1334, 1313; *anal. calc.* for C_17_H_14_Cl_2_N_6_O_2_: C 50.39%, H 3.48%, N 20.74%; found: C 50.62%, H 3.47%, N 20.83%.

*Data for 2-chloro-N-(2-(5-(((dimethylamino)methylene)amino)-1,3,4-oxadiazol- 2-yl)-6-methylphenyl)nicotinamide* **(9c).** White solid; yield, 93%; m.p. 231-232 ^o^C.^1^H-NMR (500 MHz, DMSO-*d*_6_): *δ* 10.37 (br s, 1H, -CH_3_CON*H*- ), 8.53-8.54 (m, 2H, 6-pyridine-H + =CH), 8.33 (dd, 1H, ^4^*J*_HH_=1.75 Hz, ^3^*J*_HH_=7.45 Hz, 4-pyridine-H), 7.82 (d, 1H, *J*_HH_=7.45 Hz, Ph-H), 7.61 (dd, 1H, ^4^*J*_HH_=5.15 Hz, ^3^*J*_HH_=7.45 Hz, 5-pyridine-H), 7.51 (d, 1H, ^3^*J*_HH_=7.45 Hz, Ph-H), 7.42 (t, 1H, ^3^*J*_HH_=7.45 Hz, Ph-H), 3.17 (s, 3H, -NCH_3_), 3.06 (s, 3H, -NCH_3_), 2.38 (s, 3H, -Ph-CH_3_). ^13^C-NMR (125 MHz, DMSO) *δ*: 168.11, 164.28, 159.42, 158.99, 150.97, 147.10, 138.54, 138.07, 133.76, 133.22, 128.17, 126.59, 123.50, 123.16, 41.00, 34.81, 18.66. IR (KBr, cm^-1^): *ν* 3257, 2924, 1681, 1643, 1633, 1556, 1537, 1517, 1421, 1361, 1298; *anal. calc.* for C_18_H_17_ClN_6_O_2_: C 56.18%, H 4.45%, N 21.84%; found: C 56.62%, H 4.14%, N 21.32%.

*Data for 2-chloro-N-(2-(5-(((dimethylamino)methylene)amino)-1,3,4-oxadiazol- 2-yl)phenyl)nicotinamide* **(9d).** White solid; yield, 90%; m.p. 221 ^o^C.^1^H-NMR (500 MHz, DMSO-*d*_6_): *δ* 11.30 (br s, 1H, -CH_3_CON*H*- ), 8.43-8.57 (m, 3H, 6-pyridine-H + Ph-H, =CH), 8.20 (t, 1H, *J*_HH_=6.85 Hz, Ph-H), 7.91 (d, 1H, *J*_HH_=7.4 Hz, Ph-H), 7.56-7.60 (m, 2H, 4-pyridine-H + Ph-H), 7.35 (d, 1H, ^4^*J*_HH_=2.85 Hz, Ph-H), 7.31-7.35 (m, 1H, ^5-^pyridine-H), 3.13 (s, 3H, -CH_3_), 2.99 (s, 3H, -CH_3_). ^13^C-NMR (125 MHz, DMSO) *δ*: 167.34, 164.34, 159.71, 159.33, 151.81, 147.05, 138.91, 136.52, 132.93, 132.10, 127.96, 125.41, 123.95, 122.06, 114.14, 41.45, 35.06. IR (KBr, cm^-1^): *ν* 3242, 3035, 2920, 1703, 1637, 1622, 1598, 1543, 1531, 1435, 1371, 1309, 1276; *anal. calc.* for C_17_H_15_ClN_6_O_2_: C 55.07%, H 4.08%, N 22.66%; found: C 55.42%, H 4.16%, N 22.49%.


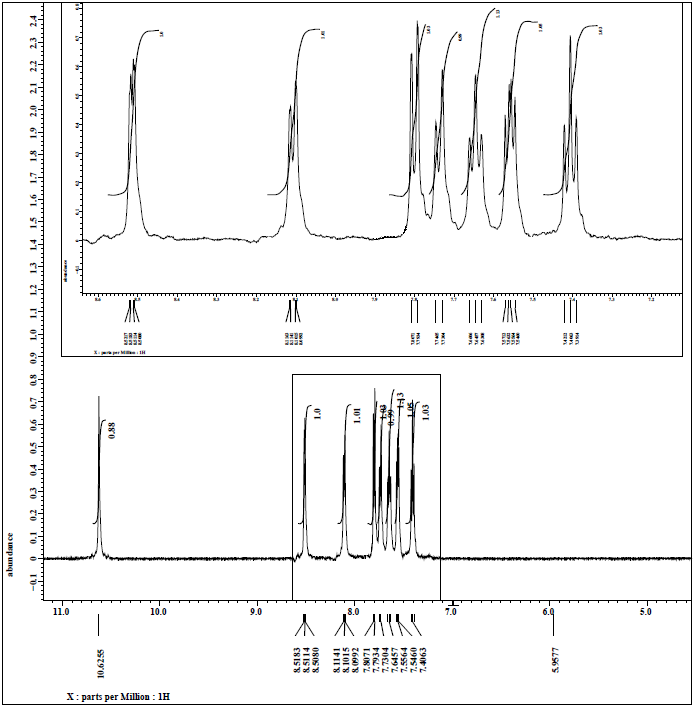


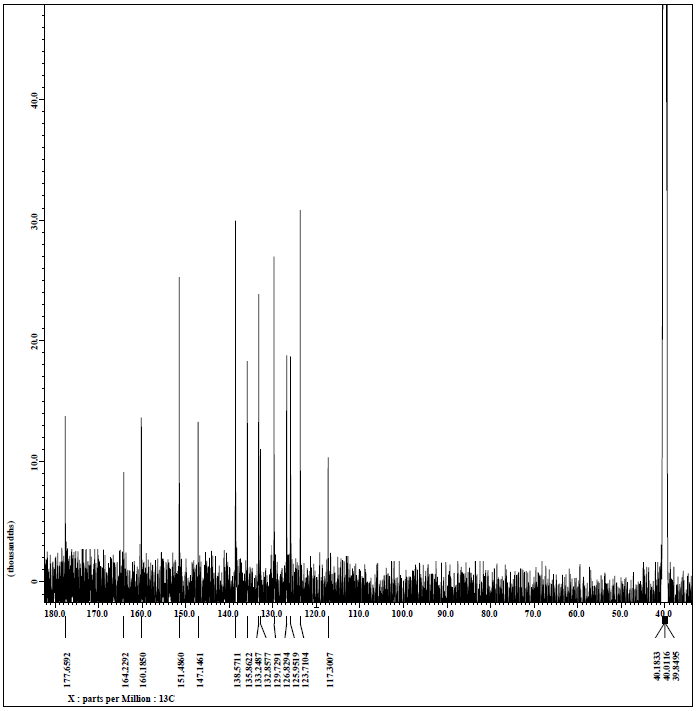


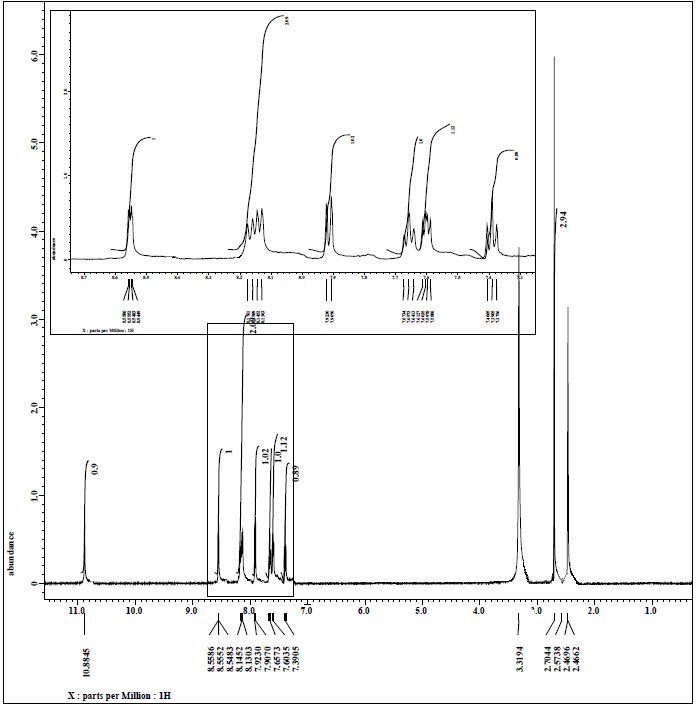


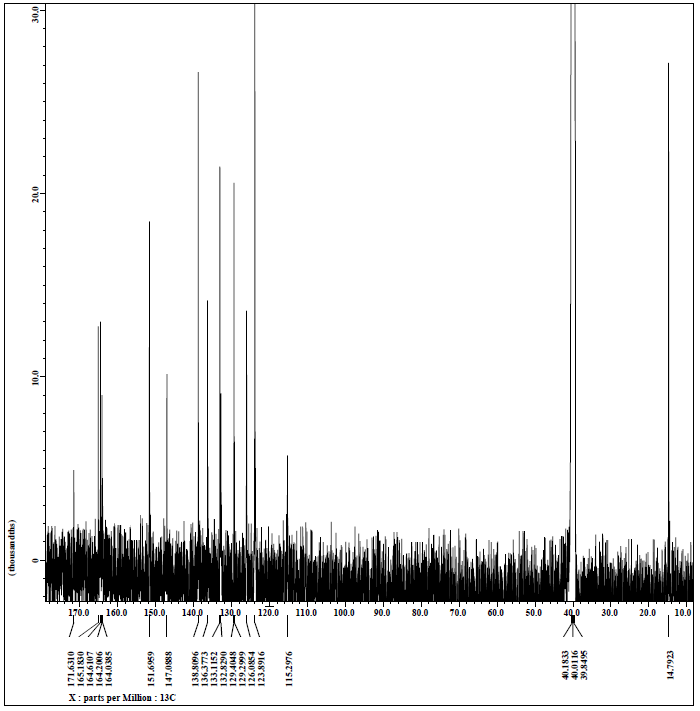


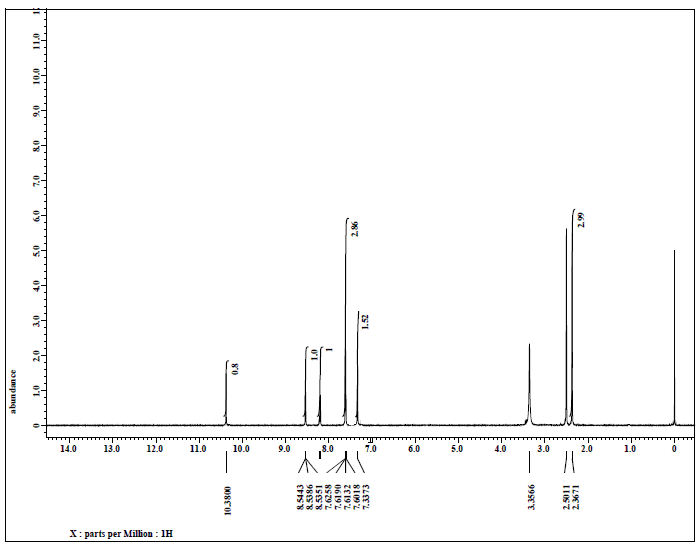


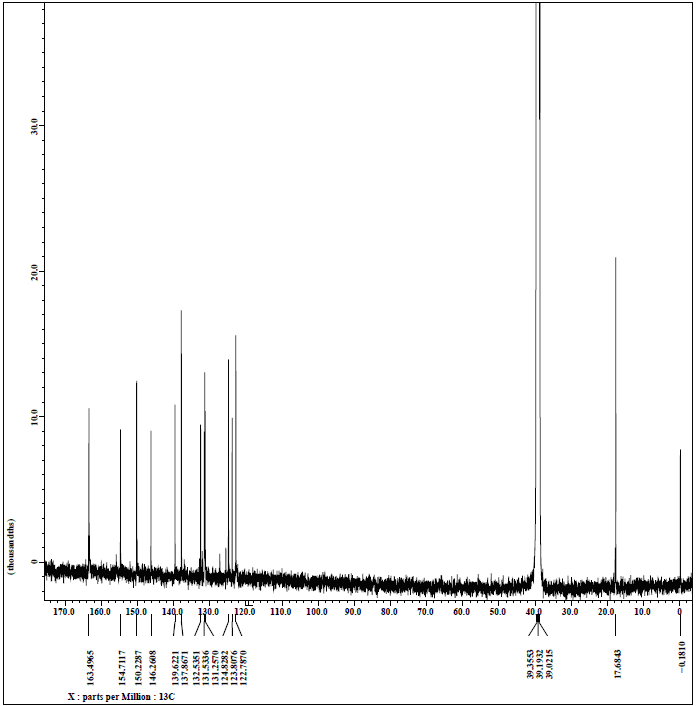


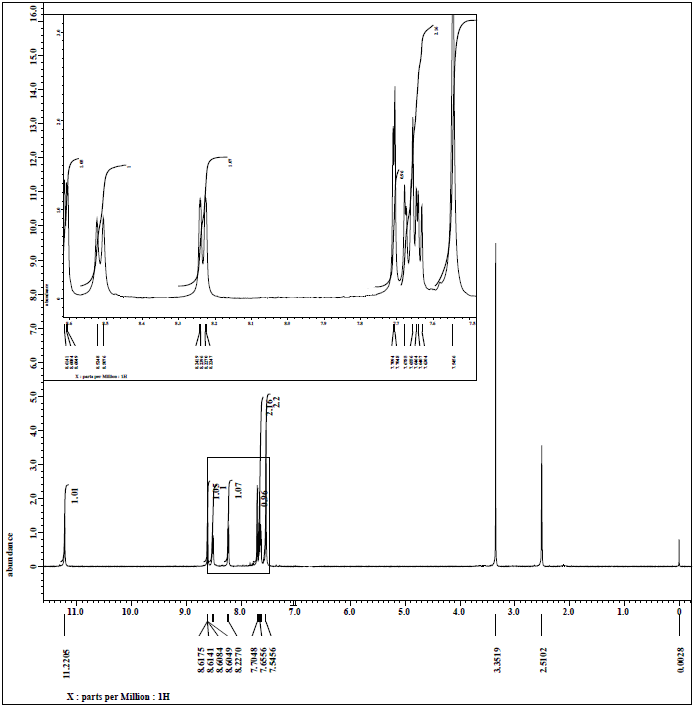


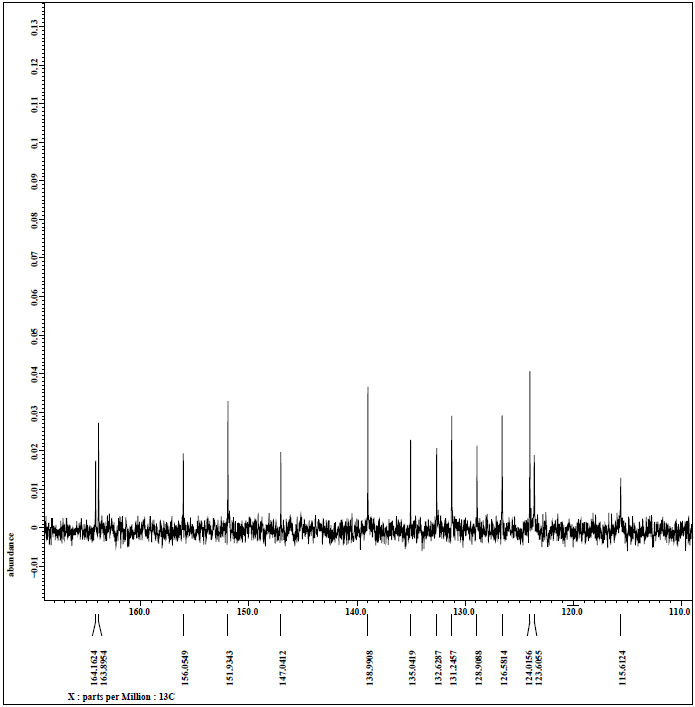


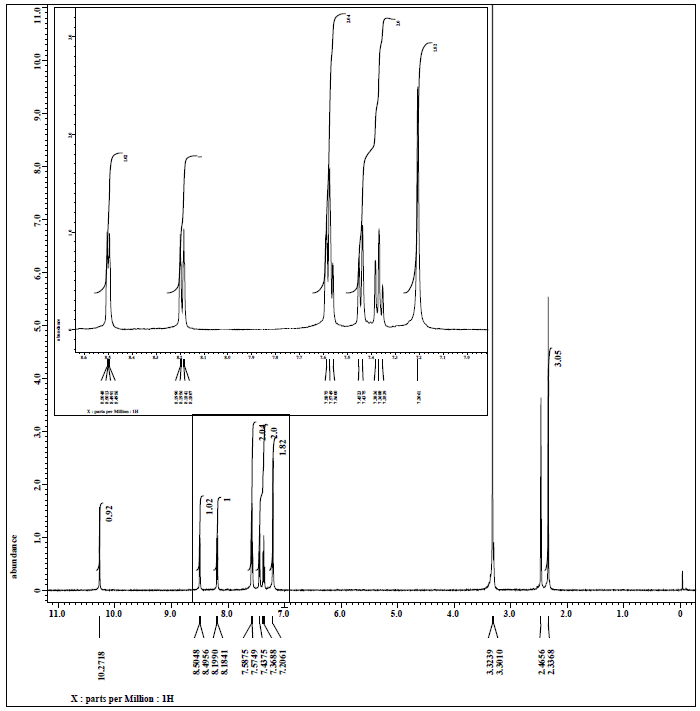


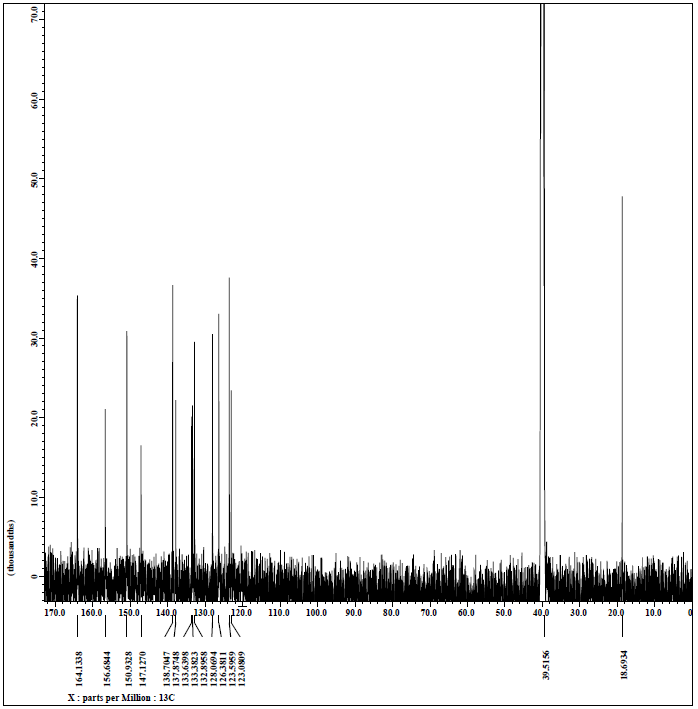


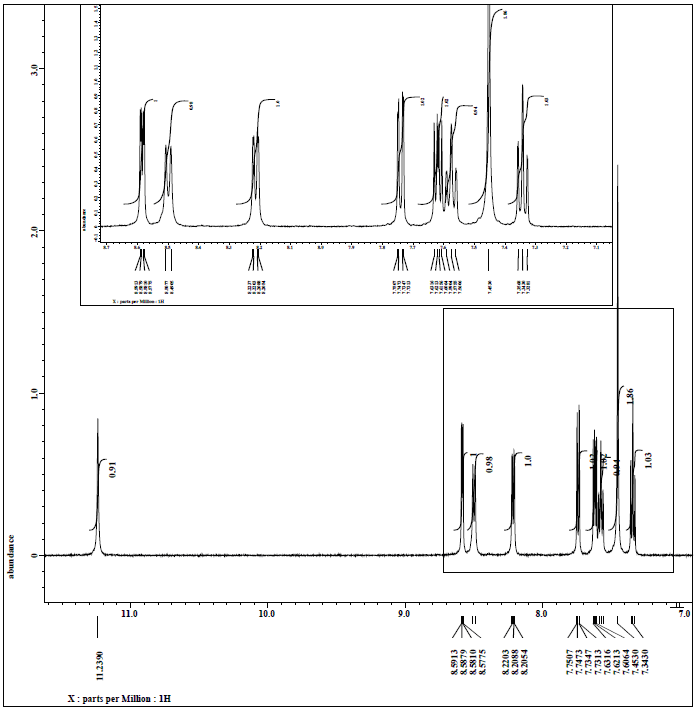


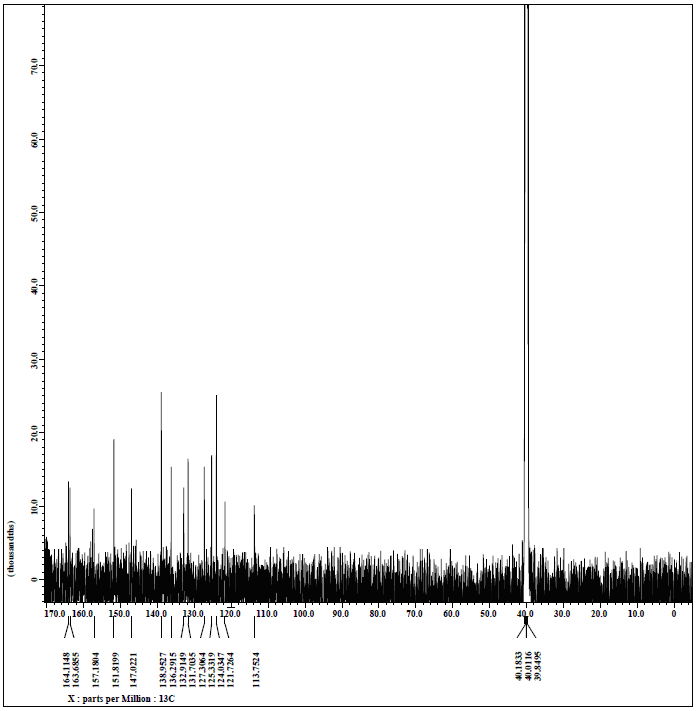


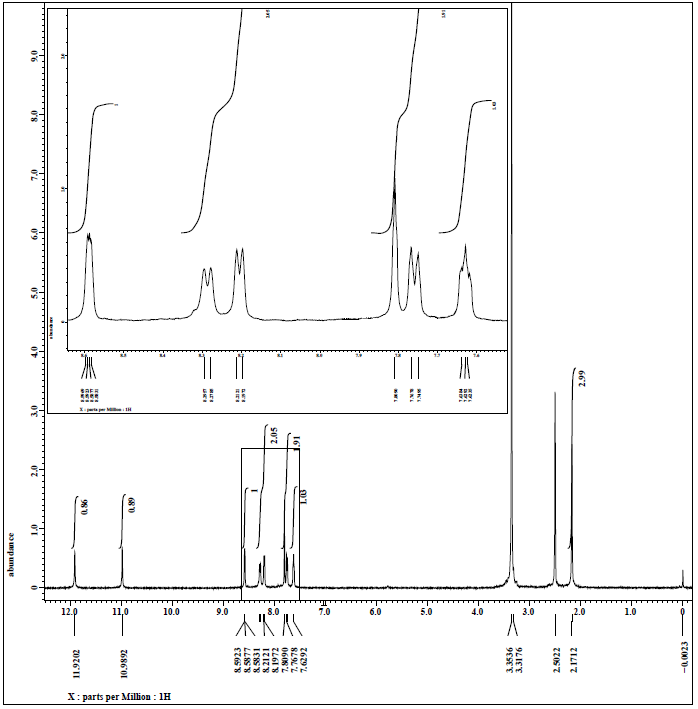


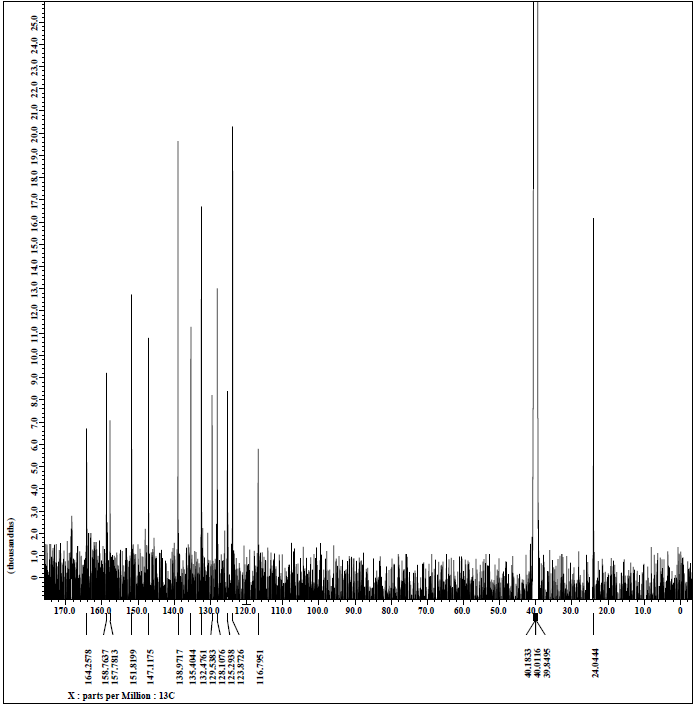


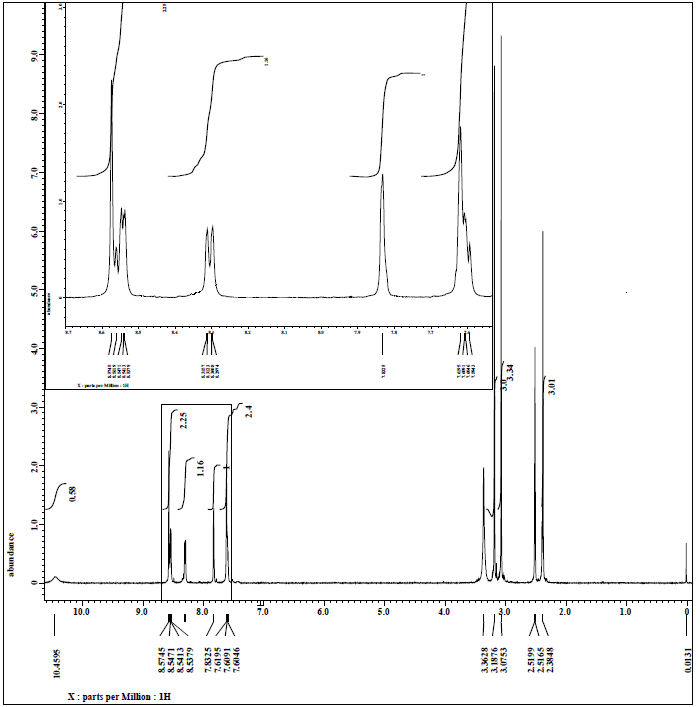


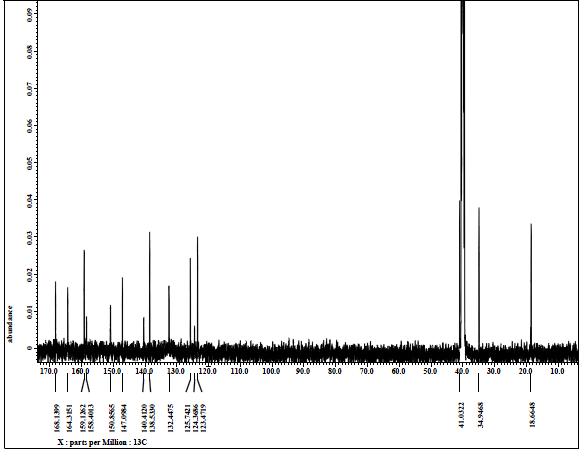


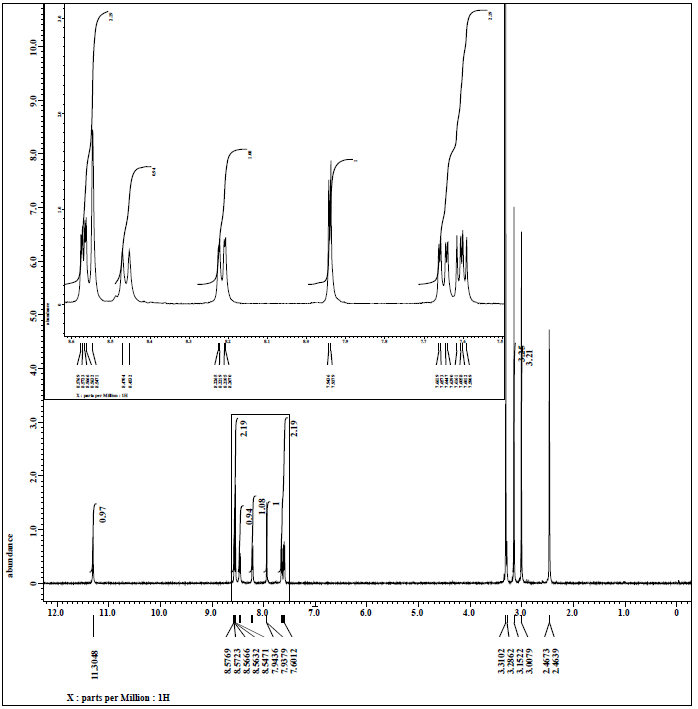


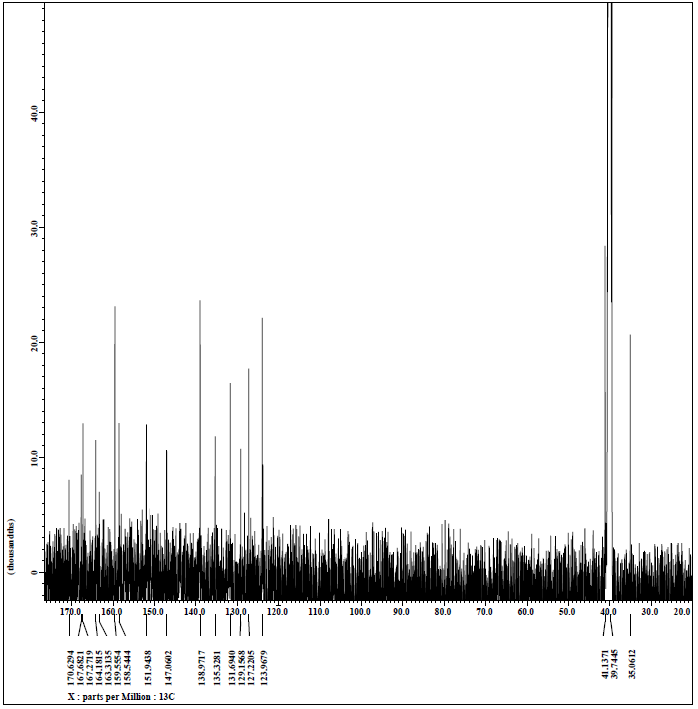


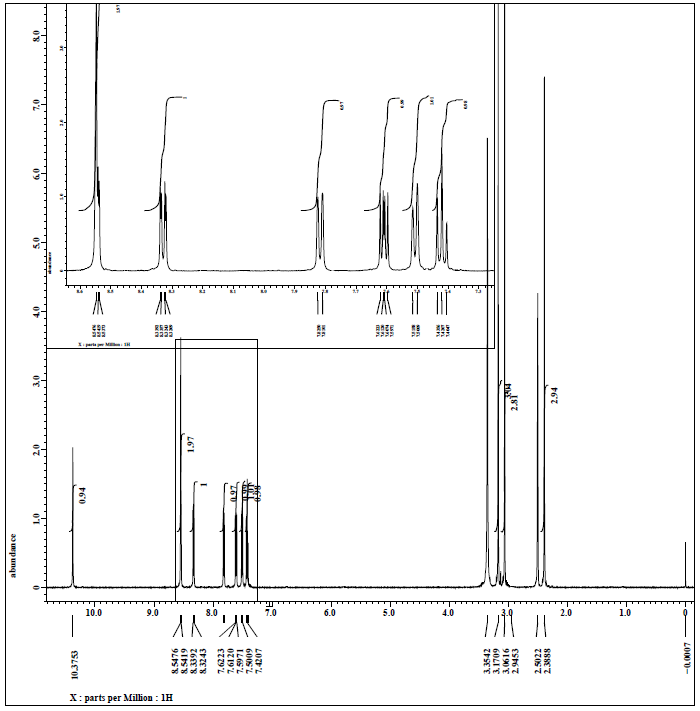


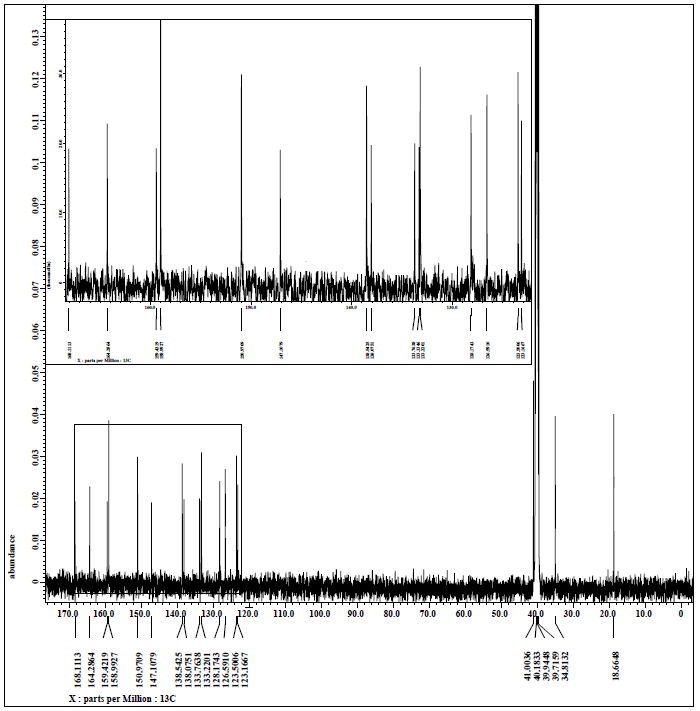


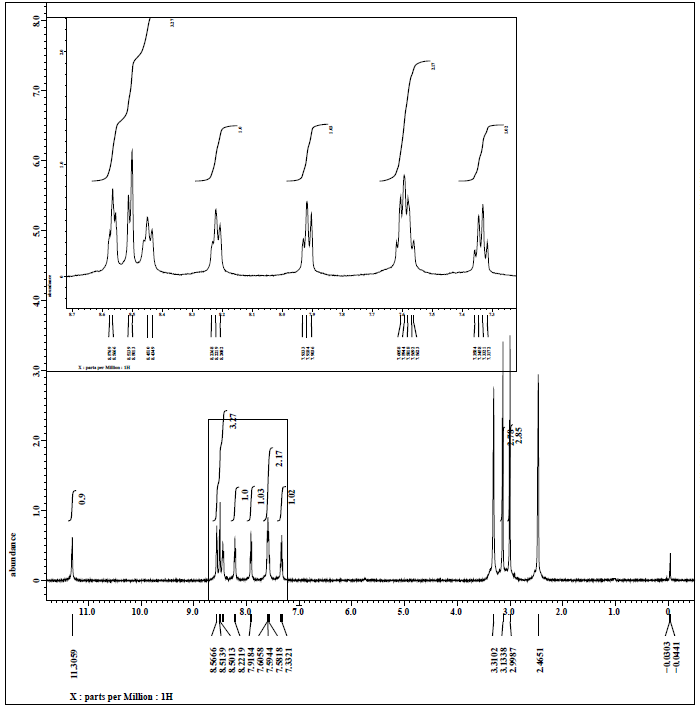


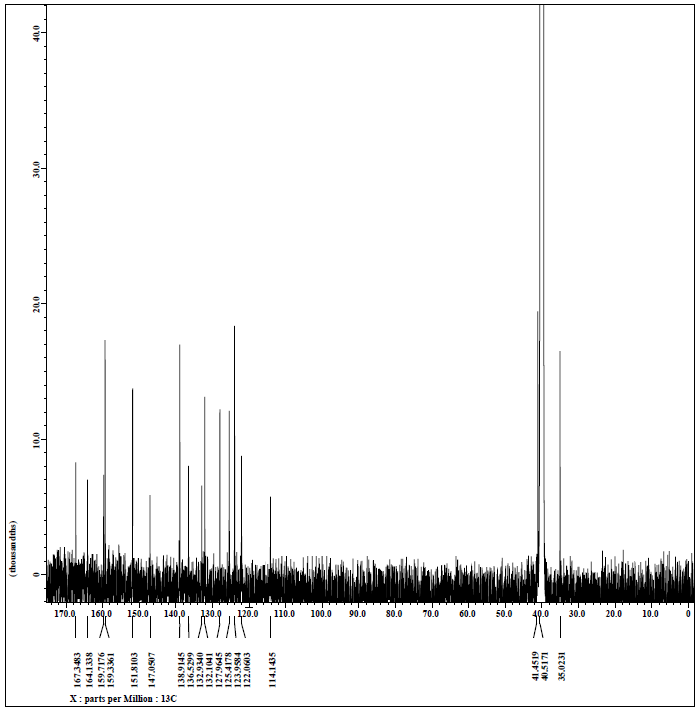

Supplement: Additional file 1 — Experimental details and spectroscopic data of the intermediates 3–4 and the title compounds 5–9. Which includes the experimental procedure and the 1H NMR of intermediates (3a-3d and 4a-4d), the experimental protocols and the spectroscopic data of title compounds 5, 6, 7a-7a, 8, and 9a-9d. All the copies of 1H NMR and 13C NMR for the title compounds were also presented in this additional file. [file 1752-153X-7-64-S1.doc]
